# *Yersinia* Species Isolated from Bats, Germany

**DOI:** 10.3201/eid1603.091035

**Published:** 2010-03

**Authors:** Kristin Mühldorfer, Gudrun Wibbelt, Joachim Haensel, Julia Riehm, Stephanie Speck

**Affiliations:** Leibniz Institute for Zoo and Wildlife Research, Berlin, Germany (K. Mühldorfer, G. Wibbelt, S. Speck); Berlin, (J. Haensel); Federal Armed Forces Institute of Microbiology, Munich, Germany (J. Riehm); 1 Current affiliation: Federal Armed Forces Institute of Microbiology, Munich, Germany.

**Keywords:** Bacteria, chiroptera, bats, zoonoses, Yersinia pseudotuberculosis, Yersinia enterocolitica, pathology, Germany, letter

**To the Editor:** Bats are distributed worldwide and are among the most diverse and species-rich mammals on earth. They exist in a large variety of distinct ecologic niches. Many bat species roost near humans, which is of particular interest for research on bat-to-human transmission of potential zoonotic pathogens. Moreover, migratory bats could act as long-distance vectors for several infectious agents. In recent decades, scientific interest in chiropteran species has markedly increased because bats are known hosts to zoonotic agents, such as henipaviruses, Ebola virus, and severe acute respiratory syndrome (SARS)–like corona viruses (*1*,*2*). However, investigations regarding bacterial pathogens with potential for mutual transmission between bats and humans are sparse. The effect of bacterial agents on individual bats is largely unknown and has been neglected in most studies published to date (*3*).

We conducted a broad study during 2006–2008 on diseases and causes of death in bats among 16 species found in Germany. Two hundred deceased bats, collected in different geographic regions in Germany (southern Bavaria, eastern Lower Saxony, eastern Brandenburg, and Berlin greater metropolitan area), were subjected to necropsy and investigated by using routine histopathologic and bacteriologic methods. During necropsy, instruments were dipped in 70% ethanol and moved into a Bunsen burner flame after every incision to prevent any cross-contamination. For bacteriologic examination, tissue samples were treated accordingly to prevent environmental contamination. A freshly cut tissue surface was plated onto Columbia agar (5% sheep blood; Oxoid, Wesel, Germany), Gassner agar (Oxoid), and MacConkey agar (Oxoid) and incubated at 37°C for 24–48 hours.

Twenty-five bacterial genera were cultured from bats, including 2 known human-pathogenic *Yersinia* spp., i.e., *Y. pseudotuberculosis* and *Y. enterocolitica*. The first *Yersinia* strain (Y938) was cultured from lung, heart, kidney (pure cultures), liver, spleen, and intestine (mixed cultures) of a greater mouse-eared bat (*Myotis myotis)*. This isolate was identified as *Y. pseudotuberculosis* by Api 20E (bioMérieux, Nürtingen, Germany), Micronaut-E (Merlin Diagnostik GmbH, Bornheim-Hersel, Germany), and 16S rRNA gene analysis (Table). The sequence was deposited into GenBank under accession no. FN561631. Further serologic characterization by agglutination test (Denka Seiken, Tokyo, Japan) and multilocus sequence typing (*4*) identified *Y. pseudotuberculosis* serogroup 1, biovar 5, sequence type (ST) 43 in all tissue samples investigated. During necropsy, severe enlargement of the liver and a marked hemoperitoneum were seen. Microscopic examination showed multifocal severe necrotizing hepatitis and splenitis associated with numerous intralesional gram-negative coccobacilli and a moderate to marked interstitial pneumonia. The remaining organs, including heart, kidney, and intestine, had no pathologic changes.

**Table:** Strain typing results of *Yersinia* spp. isolates from free-ranging bats, Germany*

Method	*Y. pseudotuberculosis†*	*Y. enterocolitica‡*
Api 20E profile§	1 0 1 4 1 1 2 1 7	1 1 5 4 7 2 3 1 7
Micronaut E,¶ % probability	100% *Y. pseudotuberculosis*	99.77% *Y. enterocolitica*
16S rRNA gene analysis, % similarity to sequences in GenBank	100% (1,058 bp) to *Y. pseudotuberculosis* YPIII#	100% (985 bp) to *Y. enterocolitica* strain 280**
Serologic characterization by agglutination test	Serogroup 1, biovar 5	Serotype O:6, biovar 1A
Multilocus sequence typing††	1 2 1 2 5 2 1	Not determined

*Y. pseudotuberculosis obtained from lung, heart, kidney, spleen, liver, and intestine; *Y. enterocolitica* obtained from spleen and intestine. For *Y. pseudotuberculosis* spleen, liver, and intestine samples, isolates were obtained in mixed culture accompanied by colonies of 1–2 nonspecific bacteria. For all remaining tissues, *Y. pseudotuberculosis* and *Y. enterocolitica* were isolated in pure culture. †GenBank accession no. FN561631. ‡GenBank accession no. FN561632. §bioMérieux, Nürtingen, Germany. ¶Merlin Diagnostik GmbH, Bornheim-Hersel, Germany. #GenBank accession no. CP000950. **GenBank accession no. FJ641888. ††Of gene loci *adk*, *argA*, *aroA*, *glnA*, *thrA*, *tmk*, *trpE.*

The second *Yersinia* strain (Y935), *Y. enterocolitica*, was isolated in pure culture from spleen and intestine of a common pipistrelle (*Pipistrellus pipistrellus*) and identified by the methods described above (Table). The 16S rRNA sequence was deposited into GenBank under accession no. FN561632. No bacteria were cultured from any other organ. Based on results of an agglutination test (Denka Seiken), the isolate was characterized as *Y. enterocolitica* serotype O:6, biovar 1A. Necropsy and histopathologic examination showed no inflammatory changes, suggesting a subclinical state of infection.

Yersiniosis is a bacterial disease with a wide distribution and host range. *Y. pseudotuberculosis* and *Y. enterocolitica* are frequently isolated from a variety of wild and domestic animals (*5*), but little is known about the occurrence of yersiniosis in free-ranging chiropteran species. Only few reports of fatal *Y. pseudotuberculosis* infection in captive flying foxes have been published (*6*,*7*). In Europe, *Y. pseudotuberculosis* strains belonging to serogroup 1 are most common and cause most *Y. pseudotuberculosis* infections in humans and animals (*5*). Isolates of ST43 in the multilocus sequence typing database (*4*) came from humans, birds, hares, hedgehog, cat, dog, and pig in Europe; humans in Asia; marsupial in Australia; and deer in Australia and New Zealand. We report an isolate from a free-ranging bat in Germany. *Y. enterocolitica* biovar 1A has been found in a wide range of human, animal, and environmental sources. Although often considered nonpathogenic, this biovar is described as an opportunistic pathogen (*8*), and serovar O:6 has been detected as the causative agent of ovine placentitis and abortion (*9*).

Transmission of both *Yersinia* species generally occurs after ingestion of contaminated food or water. All bat species in Germany are insectivorous, and insects can be infected with various microbial agents. Investigations concerning bacteria–insect interactions showed that insects may carry pathogenic bacteria, including *Yersinia* (*10*); thus, insects or contaminated water are possible sources of both species described.

In conclusion, *Y. pseudotuberculosis* and *Y. enterocolitica* were isolated from 2 bat species in Germany, representing evidence of *Yersinia* spp. in free-ranging vespertilionids. Histopathologic findings of the greater mouse-eared bat were consistent with those of systemic *Y. pseudotuberculosis* infection, rendering this species pathogenic for bats. The common pipistrelle was subclinically infected with *Y. enterocolitica*. The role of wild animals as reservoir hosts for bacterial pathogens such as *Yersinia* spp. is well known, underlining the need for biologists and persons handling wildlife to be aware of these zoonotic infectious agents.
